# Active and Passive Use of Green Space, Health, and Well-Being amongst University Students

**DOI:** 10.3390/ijerph16030424

**Published:** 2019-02-01

**Authors:** Elizabeth W. Holt, Quinn K. Lombard, Noelle Best, Sara Smiley-Smith, John E. Quinn

**Affiliations:** 1 Department of Health Sciences, Furman University, Greenville, SC 29615, USA; quinnlombard17@gmail.com (Q.K.L.); noelle.best@furman.edu (N.B.); 2 Yale School of Forestry & Environmental Studies, Yale University, New Haven, CT 06511, USA; sara.smileysmith@yale.edu; 3 Department of Biology, Furman University, Greenville, SC 29615, USA; john.quinn2@furman.edu

**Keywords:** green space, physical activity, well-being, university students

## Abstract

Frequent exposure to green space has been linked to positive health and well-being in varying populations. Yet, there is still limited research exploring the restorative benefits associated with differing types of green space use among students living in the university setting. To address this gap, we explored green space use amongst a population of undergraduate students (n = 207) attending a university with abundant opportunities to access the restorative properties of nature. The purpose of this study was to examine the type and frequency of green space interactions that are most strongly associated with indicators of health and well-being, and investigate student characteristics associated with frequent use of green space. Results revealed that students who frequently engage with green spaces in active ways report higher quality of life, better overall mood, and lower perceived stress. Passive green space interactions were not strongly associated with indicators of health and well-being. Having had daily interactions with green space in childhood was associated with frequent green space use as a university student, and identified barriers to green space use included “not enough time,” and “not aware of opportunities” These results could assist in the tailoring of “green exercise” interventions conducted in the university setting.

## 1. Introduction

A growing body of research has shown that frequent interactions with natural areas or green space are associated with positive measures of health and well-being amongst a variety of populations [1,2,3,4,5,6]. For example, indicators of health and quality of life are higher amongst those living in close proximity to parks or woodlands, building-dwellers with ample views of the natural environment from windows, and people who frequently spend time relaxing and/or being physically active in natural areas [6,7,8,9]. Students attending universities often have abundant access to green spaces, providing varied opportunities for interactions that could improve and maintain health and well-being. Yet, data characterizing the type and frequency of green space use and associated benefits conferred amongst university student populations is limited. Higher quality of life has been reported amongst students who perceive their university campus to have higher levels of “greenness” [10], and amongst university students who report higher amounts of overall time spent outdoors [11]. While these studies provide initial data suggesting interactions with green spaces could positively impact students’ quality of life, detailed data is still needed on the varying types of green space use that occur in the university setting, as well as any associated health and well-being benefits.

Previous research has shown that the mechanisms by which green space interactions could positively affect the psychological and/or physiological well-being of university students are varied. Centrally located green spaces can provide opportunities for frequent social interactions, subsequently increasing community cohesion and improving mental health outcomes [12,13,14]. Those who use green space frequently may reap psychological benefits through a more direct pathway: work from the field of Environmental Psychology suggests that immersion in a natural environment reduces exposure to the stimulating elements of everyday life and promotes “restoration” or recovery from the cognitive fatigue arising from daily stressors [15,16,17]. Thus, natural environments can serve to buffer physiological and emotional stress and also to restore attention and focus [15,16,17,18,19,20]. Finally, those who use green spaces regularly for physical activity can benefit from the many positive impacts that exercise can have on mental and physical health [21,22,23,24]. When physical activity is performed while one is immersed in a natural environment (“green exercise”) there may be additional, restorative benefits conferred [25,26,27]. Given the wide array of ways in which green space use could benefit one’s health and well-being, exposure assessments need to incorporate measures of the varying types of green space interactions, and also the “dose” (frequency and duration) of exposure [28]. Such detailed exposure data on the type and frequency of green space interactions amongst university students could help to guide the focus of campus initiatives designed to improve student well-being.

Approximately 17 million students in the USA [29] and 207 million students globally [30] attend a college or university, and a growing number of these students report that they are experiencing high levels of stress [31]. Utilizing green spaces on or adjacent to university campuses may promote restoration and consequently serve as an effective strategy to help students to buffer the stressors of college. Yet, it is not clear how often and in what ways university students are utilizing available green spaces, and very little is known regarding correlates of and barriers to frequent green space use in this specific population. In order to better understand how campus green space could be leveraged to promote and maintain student well-being, data are first needed on the types of green space interactions (e.g., active versus passive) that may be most restorative for university students. To address this gap, we conducted an exploratory study amongst a population of undergraduate students attending a university with abundant access to green space. The purpose of this study was twofold: (1) to examine the type and frequency of green space interactions most strongly associated with indicators of health and well-being, and (2) to investigate determinants of and barriers to frequent green space use.

## 2. Materials and Methods

### 2.1. Study Area, Survey Development, and Survey Administration 

**Figure 1:**
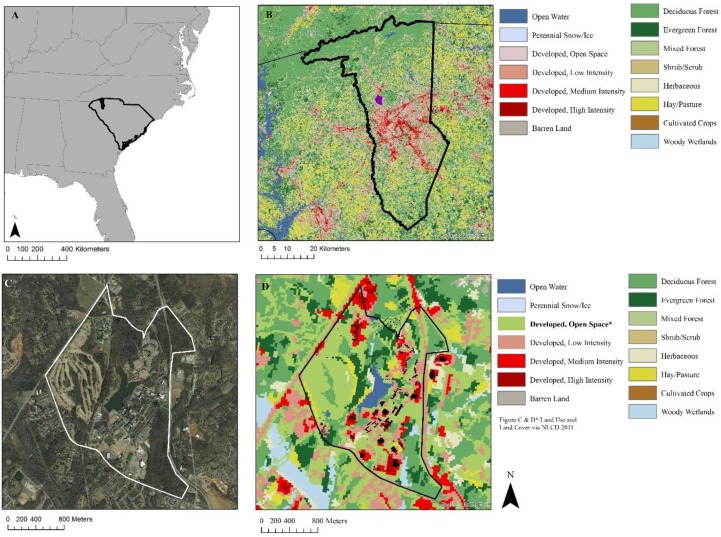
(**A**) Location of the study area (university) in the Southeastern USA with Greenville County in solid black, (**B**) Land use and land cover classification from the National Land Cover Database (NLCD 2011) for Greenville, SC, USA (black outline); purple polygon represents Furman University campus, (**C**) Aerial image of the campus (white outline), and, (**D**) campus scale NLCD 2011 image modified to show areas with <49% impervious surface (e.g., a golf course) as green space.

The study was conducted at a primarily undergraduate liberal arts university located in a suburban area of the Southeastern USA (undergraduate enrollment in year of survey = 2797). The campus covers approximately 750 acres that include a mix of open and wooded green space (Figure 1) as well as wooded areas with walking and running trails, a lake surrounded by a paved path, outdoor benches and tables overlooking water features, an outdoor amphitheater, well-maintained sidewalks between buildings, and a ¼ acre garden. A wooded mixed-use bike trail running through the surrounding town can be accessed from the campus on foot. Study investigators developed an electronic survey assessing student characteristics, frequency and type of student interactions with green space, barriers to green space use, and perceived psychosocial and physical health. An iterative process was used to develop the survey tool: drafts were reviewed by content experts, and feedback on content, design, and electronic administration was incorporated into the final survey and protocol. The final survey, consent form, and study protocol were approved by the University’s Institutional Review Board (Furman University IRB Study number 062117) and were administered using Qualtrics software (version 2.891s, Qualtrics Labs, Inc., Provo, UT, USA, 2017). In order to maximize the response rate, minimize bias, and draw a representative sample of the university population, participating classroom instructors administered the electronic survey during class time. Faculty in 11 undergraduate courses representing a range of majors and class years invited students (n = 224) to use their personal devices to open an individualized link to the consent form and survey. Students who wished to participate in the survey signed the informed consent electronically, and proceeded to open the survey. Any student who did not complete the survey in class received a follow-up email with a link to their unfinished survey. All surveys were completed in the fall semester, between 10 April 2017 and 11 September 2017.

### 2.2. Study Measures

The survey tool contained questions assessing students’ use of green spaces in and around their university campus. Because we felt that study participants could have different interpretations of the term “green space,” a standard green space definition was first identified. The research team reviewed the literature to compile a list of green space definitions used in previously published studies. Then, they asked a sample of 15 university students to rank these definitions in order of relevance to the university setting. The highest ranked definition of green space was, “Area(s) containing elements of living systems that include plants and animals across a range of scales and degrees of human management, from a small urban park through a relatively ‘pristine wilderness’” [32]. This definition was considered by study investigators to be appropriate for use in the university setting, as it covered all types of green space use from wooded trails to use of a golf course available to students. All survey questions referencing green space contained a hyperlink to this definition. 

Students’ use of green space was measured in two ways: Active use of green space was assessed by asking students how often they were physically active in green space for more than 15 min a day (over the last month), and high active use was defined as ≥4 times per week. Similarly, passive use of green space was measured by asking students how often they participated in non-physical activities in green space for more than 15 min a day (over the last month), and high passive use was defined as ≥4 times per week. Prior to these questions, students were shown pictures of campus green spaces being used for a variety of activities, and were prompted to consider how they use green space, both for physical activities (running, hiking/walking, biking) and non-physical activities (sitting, studying, relaxing with friends, meditation). In order to compare our results with previous research and provide a global assessment of students’ overall time spent outdoors, we also used a previously published “green user scale” [11]. This scale assessed students’ frequency of time spent in 9 different activities conducted outdoors, on campus (walking to and from class, exercising, participating in organized sports, socializing with friends, club meetings, studying, eating, relaxing, and working). A score of ≥30 on this scale was used to indicate frequent time spent outdoors [11,33]. 

Survey questions assessing demographic characteristics and attitudes about and experiences with natural areas were used to identify student characteristics associated with frequent green space use. Students were asked how busy they were with (1) schoolwork and (2) work or extracurricular activities, and responses were classified as very busy versus all other responses. Because prior research has shown that use of green space as a child can shape attitudes about nature and activity patterns as an adult [34,35,36] childhood green space use was assessed for structured (i.e., organized sports) and unstructured (i.e., playing in a yard, woods, or park) activities. Students were then classified according to whether or not they were “daily green space users” as a child. A student was designated as “valuing nature” if he or she responded “agree” or “strongly agree” to the question “being connected with nature is a necessity for human beings.” Students generated key words that they associated with green space, and were asked if they visited green spaces to reduce stress. Additional questions assessed barriers to green space use: students were asked whether they wished they visited green space more often, and what their primary barriers to visiting green space were.

Quality of life was measured via two questions adapted from previous studies conducted among undergraduate populations [11,37]. Students were asked, “Overall, how would you rank the quality of your life?”, and, “All things considered, how did you feel in the last 7 days?” Responses were initially rated on a 5-point Likert Scale and then dichotomized as “very satisfied” versus all other responses (high quality of life) and “very happy” versus all other responses (very happy last week). Perceived stress was measured using the 10-item Perceived Stress Scale [38], which has previously been shown to be a valid and reliable tool for assessing perceived stress in university students [39]. A student was designated as having low perceived stress if he or she scored a 12 or below, which corresponded to the lowest tertile for the sample. Self-rated general health was measured on a 5-point Likert scale and student responses were further classified as health is excellent or very good versus all other responses [40,41]. “Making health a priority” was defined by answering “agree” or “strongly agree” to the question “health is always the most important consideration when I arrange my daily activities.”

### 2.3. Statistical Analysis

Frequencies of student characteristics, attitudes about and familiarity with nature, and measures of health and well-being were calculated for the entire study population and then by high versus not high use of green space (for active, passive, and overall green user score). Then, Chi-Square tests were used to determine the statistical significance of differences. Bivariate associations were considered to be statistically significant at the *p* < 0.05 level. We were interested in understanding the type and frequency of green space interactions (active use of green space, passive use of green space, and overall green user score) that are most strongly associated with indicators of well-being (feeling “very happy”, low stress, and high quality of life). Therefore, we used separate multivariable logistic regression models, adjusted for gender, age, and students’ level of perceived “busyness”, to assess the relationship between each measure of well-being and each outcome (modeled as binary variables). For each outcome and each measure of well-being, we calculated an adjusted Odds Ratio (OR) and 95% Confidence Interval (CI). All statistical analyses were performed using SAS version 9.4 (SAS Institute, Cary, NC, USA).

## 3. Results

### 3.1. Sample Characteristics and Green Space Interactions 

Of the 224 students invited to participate in the e-survey, 220 agreed to participate. Of these, 207 provided complete information on all questions regarding green space utilization and thus were included in analyses. Characteristics of the study population and the full distribution of responses to key measures of health and well-being are shown in Table S1. The majority of students were freshmen or sophomores (74.4%), white (85.5%), and female (69.6%). Participants represented a wide range of majors across the university including the social and natural sciences, business, visual and performing arts, and the humanities. When asked to rate how busy they are on an average week during the semester, 55.6% of students were “very busy” with school work, and 37.3% were “very busy” with work or extracurricular activities. Of study participants, 58.6% reported that they were “very satisfied” or “satisfied” with their overall quality of life (High QOL), 17.2% reported that they felt “very happy” last week, 56.5% rated their overall general health as “excellent” or “very good”, and 49.5% reported they make health a priority in their daily activities. Results from scales measuring students’ utilization of green space showed that 33.3% were high active green spaces users, 33.8% were high passive green space users, and 51.7% had high overall green user scores. When asked to name their favorite green space to visit on campus for any type of activity, over half (62%) of students mentioned an activity that was related to water (relaxing by, walking or running around the lake, sitting by the koi pond). A majority (54.2%) of students reported that they visit green space to alleviate stress, and 72.4% answered “agree” or “strongly agree” that nature is a necessity for human beings (value connecting with nature). When asked to choose words that they associated with green space, the majority of students chose positive responses (77.3% chose “adventure” and 66.2% chose “feeling energized”). When asked how often they had interacted with green space as a child 53.4% and 42.1% reported having had daily, structured interactions and daily unstructured interactions, respectively. Finally, the large majority (81%) of students indicated that they wished they visited green space more often. When asked to identify their primary barriers to green space use, 71% responded “not enough time”, and 24.6% responded, “not aware of on-campus or off-campus green space opportunities”. 

### 3.2. Characteristics Associated with Frequent Green Space Use

Table 1 shows participant characteristics according to high versus not high active use of green space, passive use of green space and overall green-user score. High active use of green space was significantly associated with having had daily interactions with green space for structured and unstructured activities as a child, male gender, white race, and report of “making health a priority,” and “excellent or very good health.” High passive use of green space and high overall green-user score were each significantly associated with having had daily, structured interactions with green space as a child; however, no other characteristics were associated with overall green user score or passive green space use. Table 2 shows measure of well-being according to high versus not high active use of green space, passive use of green space and overall green-user scores. High active use of green space was significantly associated with low perceived stress, high quality of life, and feeling “very happy” last week. High passive green space use was not associated with any of the measures of well-being assessed.

### 3.3. Models

**Figure 2:**
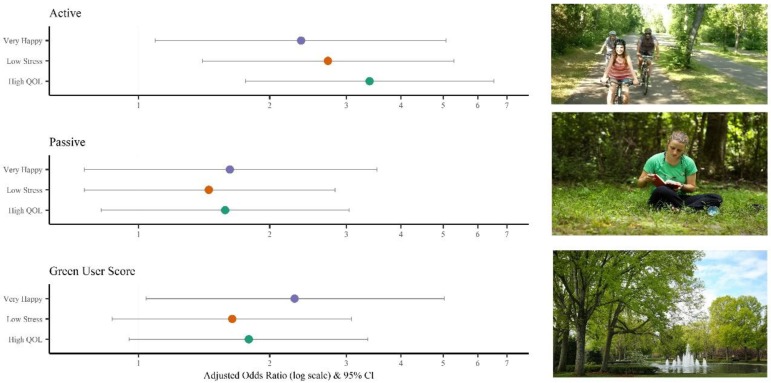
Adjusted ^☨^ Odds Ratios (95% CIs) for measures of well-being associated with students’ Active Use of Green Space, Passive Use of Green Space, and Overall Green-User Score. ☨ Odds ratios are adjusted for gender and race and students’ perceived “busyness”; High Active Use = physically active for ≥15 min in an on-campus off-campus green space ≥4 times per week; High Passive Use = sit, study, or eat for ≥15 min in an on-campus or off-campus green space ≥4 times per week; High Green User Score = Score of ≥30 on overall green user scale measuring frequency of time spent in outdoor activities.

Adjusted Odds Ratios and 95% Confidence Intervals for the associations between green space use and measures of well-being are shown in Figure 2. Relationships between students’ frequency and type of green space use and their reported happiness, stress, and quality of life were varied. A strong and statistically significant association was detected between active use of green space and each measure of well-being assessed (adj. OR = 2.36, 95% CI = 1.09, 5.07 for feeling “very happy” last week, adj. OR = 2.72, 95% CI: 1.40, 5.29 for low perceived stress, and adj. OR = 3.39, 95% CI = 1.76, 6.53 for high quality of life). High passive used of green space was not associated with any of the measures of well-being, and a high green user score was associated with feeling “very happy” last week (adj. OR = 2.28, 95% CI = 1.04, 5.02) but with not with low stress or high quality of life.

## 4. Discussion

Many university campuses provide access to well-maintained areas of green space, giving students ample opportunities to benefit from the restorative properties of green spaces via both active and passive activities. Our findings from this exploratory study of undergraduate students show not only that the frequency and type of green space use varies by key characteristics, but also that associations with health and well-being differ according to the type of utilization. Students with frequent, active interactions were more likely to report high quality of life, low stress, and feeling “very happy” last week, and the adjusted odds ratios for these associations were consistently high (>2.0) for each measure of well-being assessed. Surprisingly, passive interactions with green space (sitting, studying, eating, or socializing in a natural setting) were not associated with well-being among university students, though they did show a trend in the positive direction. 

### 4.1. Characteristics Associated with Use of Green Space by University Students

Data from this study add important information on type and frequency of green space use amongst university students. It is clear from previous research that university students value an attractive campus environment as an integral part of the university experience [42]. Our results corroborate this finding: the majority of students in this study held favorable views regarding nature/green spaces, reported that they value connecting with nature and chose words such as “adventure” and “feeling energized” to describe their attitudes about green space. Yet, only one-third of our sample reported having frequent active and one-third reported having frequent passive interactions with green spaces. Our analyses revealed key characteristics that were associated with frequent active, but not frequent passive, use of green space use, including white race, male gender, “making health a priority,” and self-rated “excellent or very good health.” While it was not surprising to see that active use of green space is associated with the markers of health, further exploration is needed to understand the observed sociodemographic differences. 

In our study, there was a strong and consistent relationship between having had daily, structured interactions with nature as a child and frequent use of green space as a university student for all types of interactions assessed (active, passive, and overall). These results are in accordance with previous research on the role that exposure to nature in childhood can play in the formation of patterns of behavior in adulthood. Among adults, attitudes about nature as well as comfort spending time in nature are linked to the amount of time spent interacting with woodlands, plants, and animals as a child [35,36]. Thompson and colleagues noted that adult patterns of green space utilization have been closely linked to patterns established in childhood, and that lack of experience with nature as a child may act to inhibit whether one seeks out opportunities to interact with green space as an adult [34]. Given recent trends showing a decline in “outdoor play” activities among USA children [43,44] we decided to separate childhood interactions with nature into “unstructured” (hiking, swimming in a creek, playing on a playground) and “structured” (organized sports such as soccer practice) activities. Our results showed that both of these types of childhood interactions were associated with active use of green space in the university setting. The varying roles that childhood interactions with the outdoors (i.e., “wilderness play” versus outdoor, structured activities) can play in influencing adult behaviors is an interesting area of research that should be explored further using longitudinal data.

### 4.2. Active versus Passive Use of Green Space and Student Health and Well-Being

Our data from a sample of university students show that frequent, active use of green space is associated with higher quality of life, lower stress, and higher self-rated general health. These findings are supported by a body of observational and experimental research linking outdoor physical activity to positive health and well-being in other populations [3,6,25,26,27,28,29]. For example, in a meta-analysis which pooled data from 10 studies, Barton and Petty showed that physical activity performed in natural settings can positively impact mood and self-esteem, and that this benefit occurs irrespective of duration, intensity, location, or gender [25]. Students in this study who report frequent time spent being physically active outdoors could be benefiting from the mood-enhancing benefits associated with exercise, the restorative properties associated with exposure to a natural environment, or perhaps most interestingly, a synergistic benefit conferred from physical activity performed in a natural setting. Our study was not designed to determine the specific mechanism by which exercising outdoors in natural areas can impact health and well-being, and thus we were unable to disentangle the relative contributions to well-being that come from exercise versus exposure to natural settings. Previous research suggests that exposure to nature could play a synergistic role in mood-enhancement during physical activity [26,45]; however, future work in this area is warranted. For example, it would be valuable to compare physical activity efforts of varying duration and intensities in a variety of indoor and outdoor environments; particularly given the popularity, heavy use, and costs of university gyms and indoor recreation centers. Future studies conducted in university populations could assess whether a lower-MET activity such as leisurely walking on an outdoor path is associated with the same health, quality of life, and mood enhancing benefits as vigorous exercise performed outdoors. Finally, an assessment of whether outdoor physical activity is performed alone, or with a friend/in a group would build upon previous research [46] and assist in understanding the additional positive effects conferred by social interactions during green exercise.

Approximately one-third of students in our study reported that they sit, study, or eat in green space four or more times per week (high passive users). However, no association was detected between passive use of green space and any of the measures of health and well-being assessed. Previous research from the field of Environmental Psychology suggests that frequent time spent outdoors in any type of activity should promote restoration and subsequently reduce stress and improve quality of life [18]. Thus, it is not clear why passive interactions with green spaces seem to be less restorative than expected among students in this study. Our data suggest that students in the university setting may need to be physically active outdoors to reap the psychological restoration that interacting with green spaces can provide. It may be that green space interactions have the greatest impact on students’ well-being when they provides an “escape” from the stressors of university life: previous research suggests that environment-oriented activities conducted in natural areas are more effective at increasing restoration than socially oriented activities [47]. It may also be true that the rigors of university life make it difficult for students to fully relax when interacting with green spaces in more passive ways. In our engaged, highly involved, and achievement-oriented university student population, a greater “dose” of passive green space may be needed for engagement with nature and related positive effects on well-being. Future research comparing the levels of restoration that students perceive to be associated with specific activities conducted in green spaces (i.e., socializing, studying, eating, and/or using social media) would add important data to the field.

### 4.3. Promoting Green Space Use in the University Setting

Recently, there have been increased efforts to implement interventions for stress reduction in the university setting, including campus initiatives such as yoga, mindfulness, and pet therapy [48,49,50,51]. Results from our study suggest that promoting opportunities for students to use green space regularly—particularly in active ways—could also be beneficial in reducing stress and improving quality of life. Many universities such as the one in this study already provide features which are known to promote green space use, including convenient access to wooded trails, well maintained natural areas, and key built environment designs such as marked paths through natural areas or around water elements [52]. Because we were interested in understanding why some students do not use or underutilize available green spaces, we assessed students’ barriers to green space use. Our results showed that students identified “not enough time” and “not aware of on-campus or off-campus green space opportunities” as primary barriers to using green space. These results are in accordance with previous research, which has shown that familiarity with or knowledge of green spaces play a key role in utilization [42,52,53,54], and that “time flexibility” and “physical activity-supporting places” are important factors in supporting green exercise [55]. Addressing students’ perceived lack of time and increasing awareness of opportunities to use green space for restoration may be important areas of focus for Universities seeking to promote health and well-being amongst students. Universities should continue to maintain paths through natural areas, while also adding educational efforts to increase students’ awareness around the location and availability of existing green spaces, and the potential quality of life benefits from regular use. Administrators, faculty and student life staff could facilitate green space use via a variety of university programming options: campus wellness programs can build familiarity by guiding students through available walking/running trails, and promote their use for stress reduction activities. University instructors could increase “active educational techniques” such as “think pair share discussions” performed while walking outdoors [56]. Finally, because data from our study showed that students hold a clear affinity for both active and passive activities performed near water features and gardens, regular use of these resources should be facilitated by providing benches and/or activity trails adjacent to such areas.

### 4.4. Strengths and Limitations

This study has many distinctive strengths when compared to prior research in this area, including a high response rate, direct measurement of varying types of green space use (active versus passive use), a standardized definition of green space that is appropriate for the university setting, and the use of previously validated scales to measure health and well-being. Our study was able to measure the actual frequency and duration of students’ interactions with green space, providing a more accurate marker of green space “dose” than previously used surrogates for green space exposure such as distance from a park or density of tree canopy cover. The sampling methods and in-class survey administration used in our study resulted in a >90% response rate, greatly reducing the potential for volunteer bias that is often a challenge in survey research. Despite these strengths, there are some limitations. The cross-sectional nature of the survey allows us to observe associations but not to assess the causal relationship between students’ use of green space and their perceived health and well-being. We recognize that the drivers of health and well-being of university students are multi-faceted, and that despite multivariable adjustment for student characteristics such as “perceived busyness”, residual confounding could still exist. Even though we carefully considered the order of survey questions and did not disclose the research hypothesis to students, some differential recall may still exist. The homogeneity of our sample reduces the potential for confounding on key characteristics such as student population and student experience (96% live on campus within walking distance of class); however, this homogeneity may limit the ability to generalize results to diverse cohorts of university students. Similarly, because this study was conducted at a primarily undergraduate university located in a suburban setting, there may be limited ability to generalize to larger universities that have a higher percentage of part-time or commuter students, and/or to urban university campuses. Additional studies of students attending universities with greater diversity in both student population and campus features (i.e., urban campuses) should be conducted. Finally, we chose to administer the survey during a period of more mild temperatures (average temperatures during the study period ranged from the mid 60s to low 70s (degrees Farenheit)). Additional data points collected over a 12-month period and/or in university settings with more extreme climates would assist in determining how patterns in student green space use vary with geography, season, and more busy or less busy periods in the academic calendar.

## 5. Conclusions

This exploratory study provides a detailed examination of green space use amongst university students: we identified not only key characteristics associated with green space use but also the specific types of interactions (i.e., active versus more passive) that are most closely associated with students’ health and well-being. Our results show that amongst students attending a university campus with abundant access to natural areas, active interactions with green spaces are associated with benefits to health and well-being, including high quality of life, low perceived stress, and feeling “very happy” last week. Our data also showed that students who regularly interacted with green spaces as children were more likely to report frequent green space use as university students, providing evidence to support the hypothesis that childhood interactions with nature can impact engagement with natural areas in adulthood.

A body of research highlights the role that exposure to and engagement with natural areas can play in the promotion and maintenance of population health: data from this study extend this research to include students living in the university setting. At many universities, students have access to campus and nearby green spaces, and regular use of these resources could serve to facilitate feelings of restoration and consequently help to buffer the stressors of college. Our results provide preliminary evidence that initiatives to increase access to and awareness of green spaces could be beneficial therapeutic interventions for stress reduction in the university setting. Such efforts would be particularly effective if they emphasize opportunities for students to integrate active use of green space into their existing routines. Future research should explore the design and testing of “green exercise” interventions conducted in the university setting.

## Figures and Tables

**Table 1:** Characteristics of the study population according to High versus Not High Active Use of Green Space, Passive use of Green Space, and Overall Green User Score (n = 207).

	Active Use of Green Space			Passive Use of Green Space			Overall Green User Score		
	High (n = 69)	Not High (n = 138)	*p*	High (n = 70)	Not High (n = 137)	*p*	High (n = 107)	Not High (n = 100)	*p*
White race, %	92.8	81.9	*	87.1	84.7		85.1	86.0	
Male gender, %	40.6	25.4	*	27.1	32.1		30.8	30.0	
“Very busy” with schoolwork, %	49.3	58.7		48.6	59.1		53.3	58.0	
“Very busy” with extracurricular activities, %	43.5	34.1		31.4	40.2		40.2	34.0	
Freshman, %	36.2	46.7		40.9	45.7		45.8	39.0	
Sophomore, %	37.7	29.0		34.3	30.7		31.8	32.0	
Junior, %	15.9	15.9		17.1	15.3		14.9	17.0	
Senior, %	10.1	9.4		2.9	13.1		7.5	12.0	
Daily, structured interactions w/green space as a child, %	66.2	47.1	**	63.8	48.2	*	65.1	41.0	***
Daily, unstructured interactions w/green space as a child, %	57.4	34.3	**	42.0	42.1		50.0	33.7	
Value connecting with nature, %	77.8	69.9		72	72.3		76.5	68.1	
Visit green spaces as a way to reduce stress, %	65.7	48.6		50.7	60.7		64.8	43.0	
Make health a priority, %	61.9	43.6	*	50	49.2		51	47.8	
Health is excellent or very good, %	87.3	42.2	**	62.5	53.7		65.7	46.9	

* *p* < 0.05, ** *p* < 0.01, *** *p* < 0.001 for chi-square tests comparing the proportion with each characteristic by high versus not high use. High Active Use = physically active for ≥15 min in an on-campus off-campus green space ≥4 times per week. High Passive Use = sit, study, or eat for ≥15 min in an on-campus or off-campus green space ≥4 times per week. High Green User Score = Score of ≥30 on overall green user scale measuring frequency of time spent in outdoor activities.

**Table 2:** Measures of well-being according to High versus Not High Active Use of Green Space, Passive Use of Green Space, and Overall Green User Score (n = 207).

	Active Use of Green Space			Passive Use of Green Space			Overall Green User Score		
	High (n = 69)	Not High (n = 138)	*p*	High (n = 70)	Not High (n = 137)	*p*	High (n = 107)	Not High (n = 100)	*p*
Felt “very happy” last week, %	27.0	12.6	*	21.9	14.9		22.6	11.5	*
Low perceived stress, %	48.6	25.2	**	39.7	32.3		39.4	29.9	
High quality of life, %	49.2	20.7	**	35.9	26.9		35.3	24.0	

* *p* < 0.05, ** *p* < 0.01, for Chi-square tests comparing the proportion with each characteristic by high versus not high use. High Active Use = physically active for ≥15 min in an on-campus off-campus green space ≥4 times per week. High Passive Use = sit, study, or eat for ≥15 min in an on-campus or off-campus green space ≥4 times per week. High Green User Score = Score of ≥30 on overall green user scale measuring frequency of time spent in outdoor activities.

## Supplementary Materials

The following are available online at http://www.mdpi.com/1660-4601/16/3/424/s1, Table S1: Characteristics of the Study Population (n = 207).
