# Supplementary material for: Active and Passive Use of Green Space, Health, and Well-Being amongst University Students
Source: Int J Environ Res Public Health. 2019 Feb 1;16(3):424. doi: 10.3390/ijerph16030424 (PMC6388138; doi:10.3390/ijerph16030424)
Supplement: Supplementary file 1 [file ijerph-16-00424-s001.pdf]

Table S1. Characteristics of the Study Population (n = 207).

Participant Characteristics	
Southeast/Gulf South Region	71.4%
Northeast Region	17.4%
Midwest/ Western Region	7.8%
Non-Hispanic White	85.5%
Black	4.4%
Latino/Hispanic	3.4%
Asian American	2.9%
Native American	1.9%
Female Gender	69.6%
Freshman	42.5%
Sophomore	31.9%
Junior	15.9%
Senior	9.7%
"Very busy" with schoolwork	55.6%
"Very busy" with extracurricular activities	37.3%
Never feel unsafe on campus	84.0%
**Green Space Use and Attitudes about/ Experiences with Nature**	
High *Active* Use of Green Space	33.3%
High *Passive* Use of Green Space	33.8%
High *Overall* Green User Score	51.7%
Daily, *structured* interactions with green space as a child	53.4%
Daily, *unstructured* interactions with green space as a child	42.1%
Being connected with nature is a necessity for human beings	
Strongly Agree	25.0%
Agree	47.5%
Neutral	21.4%
Disagree	4.1%
Strongly Disagree	2.0%
I Associate Green Space with...	
Adventure	77.3%
Feeling energized	66.2%
Boredom	14.5%
Feeling uncomfortable	7.7%
I visit green space to reduce stress	54.2%
I wish I visited green space more often	80.6%
Primary barriers to not using green space more frequently?	
Not enough time	71.0%
Difficulty accessing off-campus green space	14.5%
Not aware of off-campus green space opportunities	13.5%
Not aware of on-campus green space opportunities	11.1%
Health Risks (i.e., allergies/sun exposure)	5.8%
Other (ex: hard to use laptop outside/get wireless connection)	7.2%
**Measures of Health and Well-Being**	
When all things in your life are considered, how did you feel (most days) in the last 7 days?	

Very Happy	17.0%
Content	47.0%
OK	26.7%
Not very happy	8.1%
Miserable	1.5%
Overall, how would you rank the quality of your life?	
Very Satisfied	29.8%
Mostly Satisfied	28.8%
Satisfied	35.4%
Mostly dissatisfied	5.6%
Dissatisfied	0.5%
Cohen's Perceived Stress Scale (10-item)	
Median[IQR]	15[11,20]
Low Stress (≤12)	34.8%
Medium or High Stress(>12)	65.2%
Would you say that in general your health is…	
Excellent	22.2%
Very good	34.3%
Good	35.4%
Fair	6.1%
Poor	2.0%
Health is always the most important consideration when I arrange my daily activities:	
Strongly Agree	15.8%
Agree	33.7%
Neutral	35.7%
Disagree	12.8%
Strongly Disagree	2.0%
